# Limitations and Off-Target Effects of Tryptophan-Related IDO Inhibitors in Cancer Treatment

**DOI:** 10.3389/fimmu.2019.01801

**Published:** 2019-07-30

**Authors:** Juliane Günther, Jan Däbritz, Elisa Wirthgen

**Affiliations:** ^1^Research Group Epigenetics, Metabolism and Longevity, Leibniz Institute for Farm Animal Biology, Dummerstorf, Germany; ^2^Department of Pediatrics, Rostock University Medical Center, Rostock, Germany

**Keywords:** IDO inhibitors, immunooncology, arylhydrocarbon receptor, TRP mimetics, pharmakokinetics

## Abstract

Immunooncology is still a growing area in cancer therapy. Drugs within this therapeutic approach do not directly target/attack the tumor but interfere with immune checkpoints and target or reprogram key metabolic pathways critical for anti-cancer immune defense. Indolamine 2,3-dioxygenase 1 (IDO1) and the tryptophan (TRP)-kynurenine pathway were identified as critical mechanisms in cancer immune escape and their inhibition as an approach with promising therapeutic potential. Particularly, a multitude of IDO1 inhibiting tryptophan analogs are widely applied in several clinical trials. However, this therapy results in a variety of implications for the patient's physiology. This is not only due to the inhibition of an enzyme important in almost every organ and tissue in the body but also because of the general nature of the inhibitor as an analog of a proteinogenic amino acid as well as the initiation of cellular detoxification known to affect inflammatory pathways. In this review we provide a deeper insight into the physiological consequences of an IDO1 inhibiting therapy based on TRP related molecules. We discuss potential side and off-target effects that contribute to the interpretation of unexpected positive as well as negative results of ongoing or discontinued clinical studies while we also highlight the potential of these inhibitors independent of the IDO1 signaling pathway.

## Introduction

The degradation of tryptophan (TRP) along the kynurenine pathway (KP) plays a crucial role in the regulation of the immune response, notably as a counter-regulatory mechanism in the context of inflammation (1, 2). Three rate limiting enzymes of KP have been described thus far, tryptophan 2,3-dioxygenase (TDO2), and indolamine 2,3-dioxygenase (IDO) 1 and 2, which are regulated by both nutritional and inflammatory pathways (3). In cancers, it has been shown that an increased IDO1 activity promotes the development of an immunosuppressive microenvironment that can inhibit effective anti-tumor immune responses (4). Besides cancer cells, the tumor microenvironment included heterogeneous cell types, including endothelial cells, immune cells, and mesenchymal stromal cells (MSCs) (5). There are indications that especially MSCs contribute to a solid tumor environment by IDO-mediated immunosuppressive effects such as reducing both tumor-infiltrating T-cells as well as B-cells (6). Furthermore, studies of the microenvironment in acute myeloid leukemia demonstrate a positive correlation between increased IDO expression in MSC and elevated level of immunosuppressive regulatory T-cells (7). Therefore, the inhibition of IDO1 activity is of special interest as a target for anti-cancer therapy in order to restore tumor immunity.

In consequence, several IDO1-inhibitors are currently tested *in vitro* as well as in clinical trials (8). Many of these inhibitors such as 1-methyltryptophan (1-MT, Indoximod), INCB024360 (Epacadostat), NLG919 (Navoximod), or Norharmane are structurally related to TRP, the natural IDO substrate. These inhibitors bind to the IDO enzyme; however, they are not catabolized to N-formyl kynurenine. Frequently used IDO inhibitors in cancer therapy are Indoximod and Epacadostat which are currently under investigation in several clinical trials (https://clinicaltrials.gov). The application of IDO inhibitors should prevent both the depletion of TRP and the production of immunomodulatory TRP metabolites such as kynurenine (KYN) or kynurenic acid (KYNA) contributing to a suppression of IDO-induced immune escape of cancer cells. Although some promising results were described *in vitro* and in rodent models (9–15), however, controversy results regarding the efficiency of IDO-inhibitors were reported (16–18). This may be the result of pharmacokinetic or off-target effects such as the activation of detoxification pathways or the pretense of a nutritional signal due to TRP mimicry. This should be considered for application of TRP-related molecules *in vivo* since these effects can negatively affect the outcome of cancer treatments.

## Pharmacokinetic Aspects Affecting Efficacy of IDO Inhibitors

**Figure 1:**
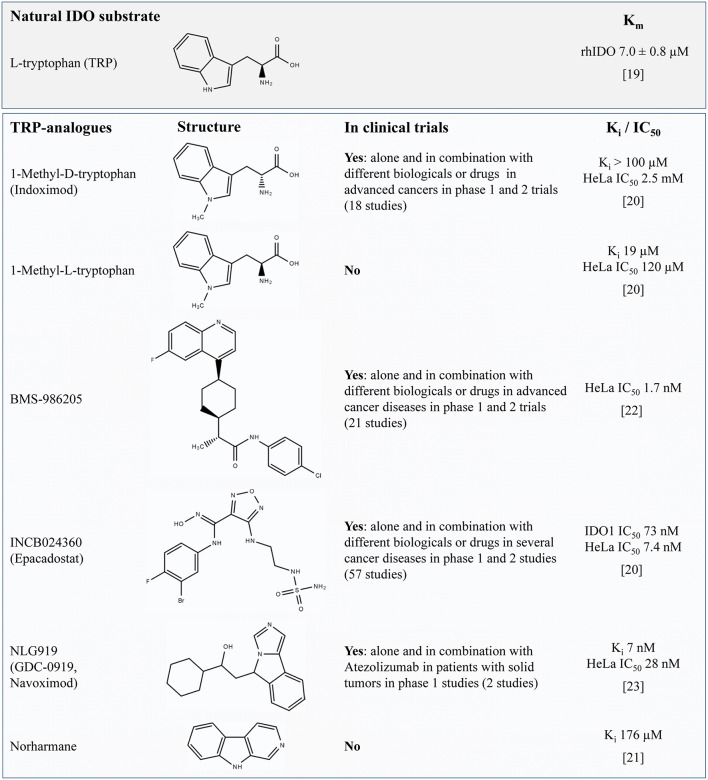
Overview of TRP-related IDO-inhibitors currently under investigation for cancer therapy in preclinical studies or clinical trials (according to their matches under https://clinicaltrials.gov, date of access: 2019-04-03). For L-TRP, one natural IDO substrate, the Michaelis constant (K_m_) is presented (defined as the substrate concentration at 1/2 the maximum velocity). The values K_i_ or the IC_50_ concentration are presented to compare the relative potency of IDO-Inhibitors. The IC_50_ value quantifies the concentration at which 50% inhibition is observed and describes the functional strength of the inhibitor under specified assay conditions (20). In contrast, K_i_ denotes the equilibrium constant of the dissociation of the inhibitor-bound enzyme complex reflecting the binding affinity of the inhibitor. It is assumed that lower values of IC_50_ or K_i_ denote a better inhibition or a tighter binding, respectively. TRP, tryptophan; IDO, indolamine 2,3-dioxygenase; K_i_, inhibitory constant.

The effective inhibition of IDO1 activity is anticipated to result in a reduced catalytic degradation of TRP to KYN and an altered metabolite profile along the KP in response to inflammatory stimuli. Most IDO1 inhibitors are synthetic TRP analogs acting as competitive inhibitors of the enzyme. To function as potent inhibitors it is necessary to reach similar or higher levels than TRP in the target tissue or to have a higher affinity to IDO than the natural substrate TRP which has a Michaelis constant (K_m_) of ~7 μM (19). Regarding the reported K_i_ or IC_50_ values shown in Figure 1 it is assumed that the TRP analogs D- and L-1-MT (21) as well as the TRP derivate Norharmane (22) act on micromolar levels while BMS-986205 (23), Epacadostat (21), and Navoximod (24) exert inhibitory effects on nanomolar levels.

### Indoximod

During last years, two stereoisomers, 1-methyl-D-tryptophan (D-1-MT = Indoximod) and 1-methyl-L-tryptophan (L-1-MT) were scrutinized as IDO inhibitors. Indoximod, which is under investigation in several clinical trials, showed therapeutic effects in murine tumor models reversing the suppression of T-cell proliferation and inducing retardation, but no total arrest of tumor growth (9, 10, 13). Unexpectedly, in an IDO1 positive ovarian cancer cell line, Indoximod triggered an increased IFNγ-induced release of the TRP metabolite KYN concurrent with an increase of IDO1 mRNA (25) indicating an activation of KP instead of anticipated inhibition. An acceleration of TRP degradation was also reported in mice and pigs after oral or subcutaneous applications of L-1-MT, which is not applied in clinical trials but used as IDO inhibitor in preclinical studies (14, 17). However, in these studies, KYNA, a stable end product of KP was increased in plasma rather than KYN, which is an intermediate metabolite of KP. However, IDO expression on mRNA or protein levels was not further investigated in these studies. Nevertheless, these results indicate that 1-MT induce an increase of TRP degradation via the KP instead of a downregulation by IDO inhibition.

One reason for the lack of effective IDO1 inhibition could be that the concentration of 1-MT was too low to inhibit IDO1 activity *in vivo*. A phase I trial of tumor patients using Indoximod as an IDO1 inhibitor has shown that doses higher than 1,200 mg 1-MT in a patient do not increase peak serum levels over ~16 μM (26), indicating a limited accumulation of the applied inhibitor. This finding is in accordance with previous findings in pigs showing that a steady-state 1-MT concentration is already reached after the second 1-MT injection of 1,000 mg/animal/day (27), increasing 1-MT to plasma levels similar to those of TRP (~30 μM). In studies using a recombinant IDO1 enzyme in cell-free assay systems determined that the L-isomer of 1-MT inhibits 50% of IDO1 activity at concentrations of 19 μM whereas the D-isomer was not effective (13). Interestingly, in mature human dendritic cells, L-1-MT only diminished IFNγ-induced increase of KYN at concentrations of 1 mM, whereas 200 μM showed no effect (28). This indicates that 1-MT was unable to prevent the production of KYN under physiological conditions in these cells which might be due to a low affinity of the inhibitor to the enzyme. Thereby, the half maximal inhibitory concentration (IC_50_) of L-1-MT and D-1-MT was 120 μM and more than 2.5 mM, respectively. As shown for 1-MT, it should be considered that K_i_ values from cell-free assays may not reflect the inhibitory effect *in vivo* resulting in inefficient inhibition at physiological concentrations. According to the reported IC_50_ values in HeLa cells it is assumed that both L- and D-1-MT are *in vivo* relatively ineffective IDO inhibitors. Nevertheless, significant effects of these drugs on immune response and KP reveal off-target modes of actions. The ineffective IDO inhibition by 1-MT *in vivo* should be considered for the interpretation of published studies using 1-MT.

### Navoximod

Navoximod is a dual specific inhibitor that inhibits IDO1 and TDO, but with only 20-fold (EC_50_ = 1.5 μM) selectivity against the later enzyme (24). Navoximod is very potent in the inhibition of IDO1 and was recently used in a phase 1 trial in combination with Azolizumab against solid tumors (29). According to the pharmacokinetics data published for orally administered doses in this study (600 or 1,000 mg), the plasma concentration of the drug should be sufficient to achieve EC_50_ inhibition of TDO. This may explain some of the effects and side effects of the treatment. The question to what extent a combined IDO1 and TDO inhibition is advantageous or disadvantageous is currently still under debate. A recently published article demonstrates the quickly absorption and moderate bioavailability of Navoximod and shows that this drug is extensively metabolized mainly by UDP-glucuronosyltransferases (UGT) (30). It would be advisable to also check the bioactivity of the resulting metabolites.

### Epacadostat

Epacadostat is described as highly potent and selective IDO1 inhibitor with moderate oral bioavailability (31). *In vitro* studies reveal that Epacadostat decreases the proliferation of regulatory T-cells concurrent with an increase of activity of cytotoxic T-lymphocytes (32). The *in vivo* IC_50_ after multiple dosing of Epacadostat is ~70 nM (33) suggesting a more potent inhibitory activity on IDO than 1-MT (21). After 100 mg oral Epacadostat application twice daily a maximum plasma concentration of 0.8 μM on day 1 and 0.9 μM on day 8 can be reached (34). It was shown that after oral application Epacadostat is metabolized in the body to IDO inactive plasma metabolites (35). The main metabolic route is glucuronidation via UGT enzymes. Another negligible primary metabolite is produced by reductive metabolism of intestinal microbiota. Further conversion of this metabolite can occur in the liver via cytochrome P450 (CYP) metabolism after absorption of this metabolite in the gut. However, data on the bioactivity of these metabolites are still missing.

*In vivo* IDO inhibition could be demonstrated in a phase I study in patients with advanced solid malignancies describing an effective normalization of serum KYN levels after oral application of Epacadostat, however, objective responses to the treatment were not observed (36). According to the promising findings that Epacadostat improves the anti-tumor response in combination with other drugs such as Nivolumab or Pembrolizumab (34, 37) the efficacy of Epacadostat in combination with various drug partners was investigated in ECHO 301–310 trials (8). However, the negative results of the phase III trial ECHO 301 in melanoma patients revealed no improvement of progression-free or overall survival by Epacadostat compared to the single treatment with the checkpoint inhibitor Pembrolizumab, which is an antibody against the programmed cell death ligand 1 (PD-L1) (38). These results raise fundamental questions about the benefits of IDO inhibition in cancer treatment. Analysis in endothelial cells of patients with advanced melanoma showed that only 17 out of 43 patients have an increased IDO expression and only 2 of 43 patients were IDO1 and PD-L1 double positive (39) revealing that IDO is not an appropriate target for the majority of melanoma patients. In this context it might be helpful to identify patients with specific immune marker phenotypes in order to develop a personalized / tailored immunotherapy.

### BMS-986205

Preclinical data reveal the potent and selective IDO1 inhibitory properties of BMS-986205 (IC_50_ = 1.7 nM in HeLa cells). Preliminary results of a clinical phase 1/2a trail with BMS-986205 alone or in combination with Nivolumab demonstrate that an IC_90_ can be reliably achieved with 200 mg daily oral application with this IDO inhibitor (23). Furthermore, it could be shown that the intratumoral KYN concentrations can be reduced by 90%. Unfortunately, there are no published data regarding the metabolization of the BMS-986205.

## Potential “Off-Target” Effects of IDO Inhibitors

### Activation of Arylhydrocarbon Receptor

There is evidence that IDO inhibitors including 1-MT (both isomers), Epacadostat, Navoximod, and Norharmane activate the ubiquitously expressed promiscuous ligand-operated arylhydrocarbon receptor (AhR) (18, 40). This is supported by the fact that Epacadostat and Navoximod are extensively metabolized by enzymes (UGT, CYP) controlled by AhR indicating that these drugs activate this signaling pathway. AhR is a ubiquitously expressed promiscuous ligand-operated receptor which mediates pleiotropic effects on the regulation of the immune response (41). As natural ligands of AhR the tryptophan metabolites, L-KYN (42), 6-formylindolcarbazole (FICZ, a photoproduct of TRP) (43) and KYNA (44), are described. After ligand-binding, AhR dimerizes with the AhR nuclear translocator (ARNT) and acts as a transcription factor, which mediates crucial effects on the pro- and anti-inflammatory regulation of the immune response (41). In this context, there is evidence that the direction of ligand-activated AhR signaling depends on the specific AhR ligand and the microenvironment (homeostatic or inflammatory) (45). In addition to transcriptional regulation, it has been suggested that AhR activation by tryptophan metabolites mediates non-enzymatic functions of IDO1. Thereby, IDO1 act as a signaling protein that contributes to TGF-β-driven tolerance in inflammatory and non-inflammatory context (42, 46). Furthermore, it is assumed that in cancer cells the AhR-mediated transcription of IL6 leads to the autocrine activation of IDO expression via STAT3 (AhR-IL6-STAT3 loop), which is associated with a poor prognosis in lung cancer (47).

The activation of AhR by IDO inhibitors may be detrimental to the assumed treatment concept of downregulating the KP by IDO inhibition. This supports several studies describing pro-carcinogenic effects of AhR ligands in several human cancers including prostate, lung, breast, pancreatic and gastric cancer (45). In transgenic mice, a constitutively active AhR induces stomach tumors (48) demonstrating an oncogenic potential of the AhR. On the other hand, there are also several reports suggesting that AhR ligands have anti-carcinogenic properties. In human colon cancer cell lines the treatment with AhR ligands such as methylcholanthrene or 3,3′diindolylmethane was able to inhibit cell proliferation and stimulate apoptosis (45). Currently, effects of prolonged AhR activation by IDO inhibitors on cancer progression are hard to predict. AhR is expressed by many cell types, including immune cells, which are important in anti-tumor response.

### TRP Mimetics in Somatic Cells

**Figure 2:**
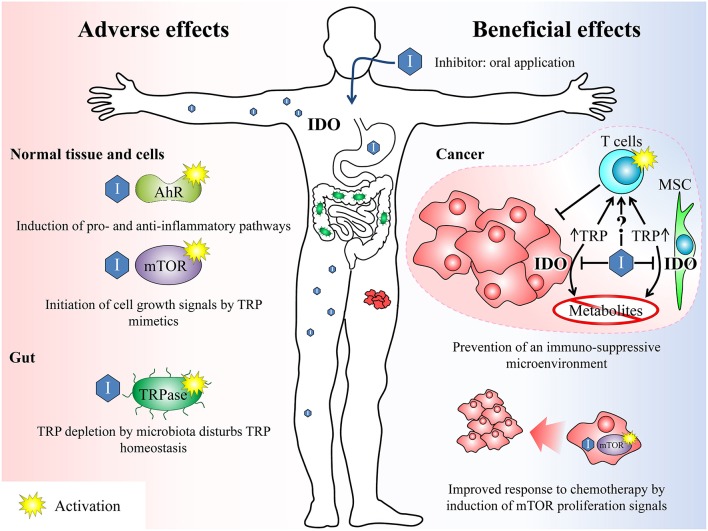
Overview of beneficial and potential adverse effects after treatment with IDO inhibitors. Beneficial effects: IDO inhibitors can break the tumour's immune escape mechanisms (immunosuppressive microenvironment) by inhibiting the TRP depletion either by inhibiting the IDO enzyme in cancer cells as well as in surrounding mesenchymal stroma cells (MSC) and thus reducing the TRP conversion or by acting as TRP mimetics. Both mechanisms can reactivate an adequate T-cell response previously suppressed by the tumor. On the other hand, the inhibition of IDO leads to a reduction of anti-inflammatory TRP metabolites, which also counteracts the formation of an immunosuppressive microenvironment. Furthermore, the activation of mTOR by TRP mimetics induces proliferation signals in the tumor. This may beneficially complement a cytotoxic anti-cancer therapy, which is most effective on proliferating cells. Adverse effects: The unspecific activation of AhR or mTOR by IDO inhibitors may induce inflammatory signaling pathways or growth signals. In the gut, TRP analogs maybe sensed by microbiota as amino acid and induce an enhanced TRP depletion by activation of the TRPase operon. TRP, tryptophan; IDO, indolamine 2,3-dioxygenase; mTOR, mammalian target of rapamycin; AhR, arylhydrocarbon receptor, TRPase, tryptophanase.

An important off-target mode of action of TRP-related IDO inhibitors is that they mimic TRP even in the context as fake nutritional signals. They may target the mammalian target of rapamycin (mTOR) signaling which is the central pathway in amino acid sensing and signaling (49). Activation of mTOR via amino acids leads to initiation of a range of cellular processes including cell growth, proliferation, differentiation, and metabolic alterations. High amounts of TRP analogs may feign an amino acid oversupply. This could potentially be dangerous because cells cannot react adequately to the amount of nutrients actually available. However, it is known that 1-MT can reactivate the mTOR activity inhibited by TRP depletion in cancer microenvironment (50). This has beneficial effects for subsequent chemotherapeutic intervention and may be the most important cause of antitumor effects of this inhibitor (16) (Figure 2). Most cytotoxic drugs used in anti-cancer therapy target highly proliferative cells. Therefore, an enhanced mTOR activity, especially in otherwise “cold” tumors, may boost the efficacy of these cytotoxic therapies. Furthermore, T-cells are important players of the host immune system against cancer. These immune cells depend on mTOR signaling to integrate danger signals for their proper activation (51). Therefore, the TRP depleted microenvironment typical for a range of tumors impairs T-cell proliferation and function. Reactivation of T-cell function via activation of mTOR may overcome the tumor immune escape and beneficially complement an anti-cancer therapy.

### TRP Mimetics in Microbiota

It has been shown *in vitro* that the 1-MT-induced increase in TRP may impede the antimicrobial and immunoregulatory functions of LPS-induced TRP depletion (52), facilitating chronic infections due to impaired pathogen growth arrest (53). During cancer therapy, this might increase the risk of chronic uncontrolled infections due to an insufficient host immune response. A problem of oral TRP mimetic application is furthermore, that subsets of enteric bacteria express TRP degrading enzymes (54). Tryptophanase is one of these bacterial enzymes which are known to be induced by TRP in the gut (55). Most TRP analogs like 1-MT are no substrate of this enzyme (56). However, they are sensed by the bacteria as amino acid and therefore equally effective induce the tryptophanase operon necessary for high enzyme expression (56). This may lead to enhanced TRP depletion in the gut by microbiota. Although, the physiological consequences of the described effects remain unknown, it can be concluded that the systemic application of TRP analogs disturbs the homeostasis of TRP metabolism which might affect cell metabolism, immune response, and growth of potential enteric bacteria. The TRP mimetic properties were extensively investigated only for 1-MT. However, it cannot be excluded that other TRP-related inhibitors may also mimic TRP and interfere with the described metabolic pathways.

## Conclusion

Several IDO1-inhibitors are currently tested *in vitro* as well as in clinical trials. However, there are controversial results regarding the efficacy of IDO inhibitors for IDO inhibition and cancer treatment. It should be considered that some competitive inhibitors such as 1-MT or Norharmane can hardly induce effective *in vivo* IDO inhibition due to their low *in vitro* potency and the fact that they reach plasma concentrations similar to those of the IDO substrate TRP. Therefore, the immunomodulatory effect of these inhibitors in the body is most likely related to off-target effects such as AhR activation or fake nutritional signaling rather than IDO inhibition. This should be considered when interpreting the significance of IDO in *in vitro* and *in vivo* studies with these inhibitors. In contrast to 1-MT and Norharmane, more potent inhibitors such as Epacadostat or Navoximod inhibit IDO on nanomolar levels resulting in decreased KYN production *in vitro* and *in vivo*. Nevertheless, the failure of a large phase III clinical trial with Epacadostat in melanoma patients indicates that IDO, at least in this type of cancer, is not a suitable target to improve the efficacy of drugs such as Pembrolizumab. In this context, it might be beneficial, for example, to use a molecular diagnostic approach such as biomarker profiles to clarify in advance to what extent IDO inhibition can be beneficial for the respective cancer treatment strategy. Furthermore, the pro- and anti-carcinogenic effects of mTOR and/or AhR activation may be a double-edged sword depending on the type of cancer, the tumor environment and the concurrent anticancer treatment. These side effects, which have so far not been considered sufficiently, should definitely be taken into account in the development of beneficial synergistic drug combinations in cancer therapy. In this context, computational approaches and bioinformatic modeling could become increasingly important in the future.

## Author Contributions

EW and JG wrote the first draft of the manuscript. JD critically revised the manuscript for important intellectual content. Each author has approved the final version of the manuscript before publication.

### Conflict of Interest Statement

The authors declare that the research was conducted in the absence of any commercial or financial relationships that could be construed as a potential conflict of interest.
